# Sub-Lethal Dose of Shiga Toxin 2 from Enterohemorrhagic *Escherichia coli* Affects Balance and Cerebellar Cytoarchitecture

**DOI:** 10.3389/fmicb.2016.00133

**Published:** 2016-02-10

**Authors:** Luciana D’Alessio, Alipio Pinto, Adriana Cangelosi, Patricia A. Geoghegan, Carla Tironi-Farinati, Gabriela J. Brener, Jorge Goldstein

**Affiliations:** ^1^Centro de Epilepsia, Hospital Ramos Mejía and Instituto de Biología Celular y Neurociencia Prof. E. De Robertis, Consejo Nacional de Investigaciones Científicas y TécnicasBuenos Aires, Argentina; ^2^Laboratorio de Neurofisiopatología, Instituto de Fisiología y Biofisica “HOUSSAY”, Consejo Nacional de Investigaciones Científicas y Técnicas, Facultad de Medicina, Universidad de Buenos AiresBuenos Aires, Argentina; ^3^Centro Nacional de Control de Calidad de Biológicos, Administración Nacional de Laboratorios e Institutos de Salud Dr. Carlos G. MalbranBuenos Aires, Argentina

**Keywords:** cerebellum, neurodegeneration, transmission electron microscopy, fluorescence microscopy, blood–brain barrier

## Abstract

Shiga toxin producing *Escherichia coli* may damage the central nervous system before or concomitantly to manifested hemolytic–uremic syndrome symptoms. The cerebellum is frequently damaged during this syndrome, however, the deleterious effects of Shiga toxin 2 has never been integrally reported by ultrastructural, physiological and behavioral means. The aim of this study was to determine the cerebellar compromise after intravenous administration of a sub-lethal dose of Shiga toxin 2 by measuring the cerebellar blood–brain barrier permeability, behavioral task of cerebellar functionality (inclined plane test), and ultrastructural analysis (transmission electron microscope). Intravenous administration of vehicle (control group), sub-lethal dose of 0.5 and 1 ηg of Stx2 per mouse were tested for behavioral and ultrastructural studies. A set of three independent experiments were performed for each study (*n* = 6). Blood–brain barrier resulted damaged and consequently its permeability was significantly increased. Lower scores obtained in the inclined plane task denoted poor cerebellar functionality in comparison to their controls. The most significant lower score was obtained after 5 days of 1 ηg of toxin administration. Transmission electron microscope micrographs from the Stx2-treated groups showed neurons with a progressive neurodegenerative condition in a dose dependent manner. As sub-lethal intravenous Shiga toxin 2 altered the blood brain barrier permeability in the cerebellum the toxin penetrated the cerebellar parenchyma and produced cell damaged with significant functional implications in the test balance.

## Introduction

Shiga toxin-producing *Escherichia coli* (STEC) causes hemorrhagic colitis which may leads to HUS (O’Brien and Kaper, 1998), resulting in symptoms which include: thrombocytopenia, microangiopathic hemolytic anemia, and acute renal failure (Proulx et al., 2001).

At present, Argentina has the highest number of HUS cases with about 420 new cases reported annually, affecting 17/100,000 children under the age of five (Rivas et al., 2010). It has been reported that the mortality rate derived from HUS ranges up to 5% of the cases, and between 7 and 40% when the central nervous system (CNS) is involved (Eriksson et al., 2001; Noris and Remuzzi, 2005; Repetto, 2005; Oakes et al., 2006; Magnus et al., 2012). Furthermore, in 2011, Europe’s largest reported STEC outbreak started in northern Germany had higher rate of neurologic complications (Trachtman et al., 2012). Probably due to a new variant, which expresses not only the potent Stx2, but also enteroaggregative elements that conferred a major toxicity (Frank et al., 2011; Weissenborn et al., 2012).

Damage in the CNS may occur before or concomitantly with other symptoms of the systemic HUS disease (Brascher and Siegler, 1981; Karmali et al., 1985). Common clinical signs of severe CNS injury included focal seizures, changes in the level of consciousness (from lethargy to coma), hemiparesis, descerebrate posture, cortical blindness, ataxia, cranial nerve palsy, hallucinations, and brain stem symptoms (Gianantonio et al., 1973; Cimolai et al., 1992; Hamano et al., 1993; Tapper et al., 1995; Hager et al., 1999). Mice models of Stx2 intoxication matched with neurological symptoms observed in human patients and others common in rodents: lethargy, spasm-like seizure, reduced spontaneous motor activity, abnormal gait, hind limb paralysis, pelvic elevation and shivering (Obata et al., 2008; Tironi-Farinati et al., 2013). In addition, studies performed by TEM revealed subtle but significant changes in the brains of animal administered with high-dose Stx, these changes included neuronal, fiber, and glial ultrastructural alterations. The principal areas affected were neocortex, cerebellum and basal ganglia (Fujii et al., 1994, 1996, 1998; Mizuguchi et al., 1996; Fletcher et al., 1999; Goldstein et al., 2007).

It has been observed that the cerebellum is frequently damaged in patients with HUS (Weissenborn et al., 2012). Most frequent neurologic symptoms included: dysdiadochokinesis, dysmetria, intention tremor, cerebellar ataxia, dysarthria, and nystagmus (Weissenborn et al., 2012). However, research in this field has been scarce requiring a more integrative approach (Mizuguchi et al., 1996; Fujii et al., 1998; Mewasingh et al., 2003). Therefore, the current work attempts to integrate the physiological, behavioral and ultrastructural implications of cerebellar functioning caused by Stx2 addressed for the first time.

## Materials and Methods

### Sub-Lethal Dose

The canonical Stx2 used was obtained from phage 933W, named Stx2a (Plunkett et al., 1999). It was purchased at Phoenix Laboratory, Tufts Medical Center, Boston, MA, USA and were checked for lipopolysaccharide (LPS) contamination by the Limulus amoebocyte lysate assay. It contained <10 pg LPS/ng of pure Stx2. Different amounts of Stx2 (5–0.44 ηg per animal) or vehicle were intravenously (i.v.) administered in mice, as previously described (Tironi-Farinati et al., 2013). Survival time was considered when 100 % of the animals survived with an administration of 1 ηg of Stx2 or less amount for at least 8 days. Also it was observed that under these dose mice did not die even at day 10. Therefore, this amount was considered sub-lethal and selected to use for the present work.

### Animals

Male NIH mice (25–30 g) were housed in an air conditioned and light-controlled (lights between 07:00 and 19:00 h) animal facility. Test animal were obtained from animal facility center from Administración Nacional de laboratorios e Institutos de la Salud (ANLIS), Malbrán, Argentina. Mice were provided with food and water *ad libitum*. They were daily monitored at the same time for neurological manifestations from the beginning of the experiments until the last day.

For all studies a set of three independent experiments were performed. Mice were divided into three groups (*n* = 6): animals treated with vehicle (control), with a sub-lethal i.v. administration of Stx2 (1 ηg per mouse), and with half of sub-lethal i.v. administration of Stx2 (0.5 ηg per mouse). The three groups were tested for behavioral and ultrastructural studies following Stx2 administration.

The experimental protocols and euthanasia procedures were reviewed and approved by the Institutional Animal Care and Use Committee of Buenos Aires University School of Medicine (Resolution N° 2437/2012). All procedures were performed in accordance with the guidelines for care and use of experimental animals (EEC Council 86/609).

### BBB Permeability Test

This test was performed, as described by (del Valle et al., 2008). Mice were divided into two groups (*n* = 4): animals were i.v. treated with saline as vehicle (control group) or with a sub-lethal administration of 1 ηg of Stx2. After their respective treatments at day 4 they were perfused transcardially with 0.9% NaCl solution followed by a solution with 4% paraformaldehyde, and 1% Evans Blue (EB) in 0.1 M phosphate buffer solution (PBS) [fixative per animal weight (ml/g)]. Cerebella were removed from the skull and post-fixed with the same fixative solution (without the EB staining) for 2 h (del Valle et al., 2008). Cerebellar coronal sections (25 μm thick) were mounted on slides with a solution of glycerol and PBS (3:1) and were examined under Olimpus confocal microscope Fluoview FV1000 (Melville, NY, USA). The staining with EB dye was visualized by excitation with 543-nm laser beams (green zone) and visualized as red fluorescence.

### Inclined Plane Test

The inclined plane test consists in a platform of 1 meter long and 30 cm wide, with an analog protractor and hinged base, elevated at 5° intervals until the animal slipped backwards (Chang et al., 2008; Lekic et al., 2011). The maximum angle at which a mouse is able to maintain its position for at least 5 seconds constitutes the inclined plane score (Wells et al., 2003). The inclined plane score was determined in animals injected with vehicle or 0.5 and 1 ηg of Stx2 at day 1, 3, 5 and 8 days following respective treatments.

### Transmission Electron Microscopy

TEM analysis was performed to study ultrastructural changes in the cerebellum following the treatments described in Section “Animals”. Mice were anesthetized with Sodium pentobarbital (60 mg/kg) and perfused transcardially with 0.9% NaCl solution followed by 2.5% glutaraldehyde in 0.1 M phosphate buffer [fixative per animal/weight (ml/g)]. Brains were removed from the skull and post-fixed in the same fixative solution for 2 h. Samples of cerebella (3 mm^2^ thick) were dissected and collected in 0.1 M phosphate buffer. The samples were first assessed by light microscopy with blue toluidine to select the areas for TEM. Ultrathin sections were cut from selected areas and then contrasted with 1% osmium tetroxide and 1% uranyl acetate, dehydrated and flat-embedded in Durcupan (Priestley et al., 1992). The sections were contrasted with lead citrate and then examined and photographed on a Zeiss 109 TEM (Jena, Germany). Adobe Photoshop software was used in the assembly of images (Adobe Systems Inc., San Jose, CA, USA).

Neuronal damage caused by Stx2 was determined by EM and consequently quantified. Neuronal damage was considered when gathered at least one of these criteria: cell edema, vacuolated cytoplasm, hypertrophic axons and/or retracted cytoplasm. Quantification of damaged Purkinje cells was determined and expressed as a percentage of the total number of Purkinje neurons in an area of 3721 μm^2^.

### Statistical Analysis

The data are presented as mean ± SEM. In the case of BBB permeability assay and damaged Purkinje cells analysis, statistical significance was performed using one-way analysis of variance (ANOVA) followed by Student–Newman–Keuls *post hoc* tests. In the case of comparison of different treatment groups at different time points in the cerebellar functionality test, two ways analysis of ANOVA was used followed Bonferroni *post hoc* test (GraphPad Prism 4, GraphPad Software, Inc.). The criterion for significance was *p* < 0.05 for all the experiments.

## Results

### BBB Permeability of Cerebellum was Increased by Stx2

**FIGURE 1:**
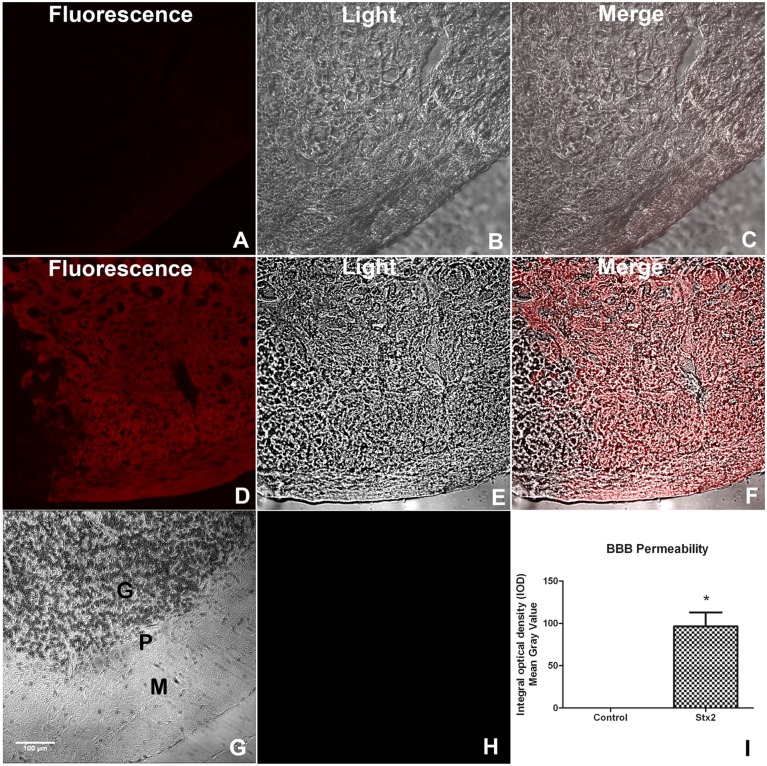
**A sub-lethal dose of Shiga toxin 2 (Stx2) increases blood–brain barrier (BBB) permeability in the cerebellum.** Evans blue (EB) staining was employed to show permeability of the BBB **(A–F)**. Saline-treated cerebellum **(A–C)**. Fluorescence micrograph failing to detect EB staining **(A)**. Light microscope micrograph showing the same saline-treated area **(B)**. Merge micrograph between micrographs **(A)** and **(B)** **(C)**. Stx2-treated cerebellum **(D–F)**. Fluorescence micrograph showing EB staining in the parenchyma of Stx2-treated cerebellum **(D)**. Light microscope micrograph showing the same Stx2-treated area **(E)**. Merge micrograph between micrographs **(D)** and **(E)** **(F)**. Hematoxylin & Eosin staining of the observed area of the cerebellum showing the three layers involved in the staining of EB: M, molecular; P, Purkinje and G, granular layers **(G)**. Negative control of a cerebellum by not adding EB **(H)**. Quantification of BBB permeability in the cerebellum **(I)**. Significant differences between toxin-treated and control group (^∗^*p* < 0.05). The scale bar in **(G)** applies to all micrographs.

It has been reported that Stx2 can pass through the BBB affecting the cell parenchyma (Goldstein et al., 2007). To determine whether the toxin changes BBB permeability the EB dye was perfused in each treated-mice group. Mice injected i.v. with vehicle showed no permeability to the EB dye (**Figures 1A-C,G,H**), indicating that the BBB was conserved. In contrast, mice injected with 1 ηg of Stx2 showed an intense fluorescence staining in the parenchyma, indicating that EB succeeded to pass through the BBB to the cerebellar parenchyma (**Figures 1D–F**). Therefore, i.v. administration of Stx2 increased the BBB permeability (**Figure 1I**).

### Cerebellar Functionality was Altered by Stx2

**FIGURE 2:**
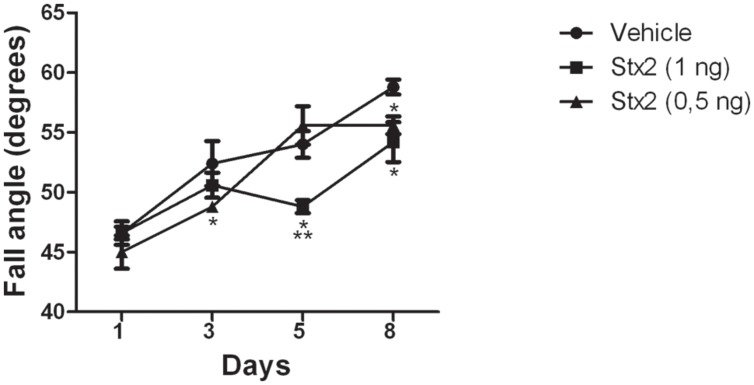
**Changes in cerebellar functionality by Stx2: quantification of inclined plane scores at different days and for different treatments.** Significant differences between toxin-treated and control groups at the same day (^∗^*p* < 0.05). Significant differences between 1 ηg toxin-treated and 0.5 ηg toxin-treated groups at the same day (^∗∗^*p* < 0.05).

As Stx2 treatment provoked a deleterious effect on the BBB by increasing its permeability we asked whether the toxin may also alter cerebellar functionality. For that purpose, a behavioral test was made to evaluate the sensory-motor function in combination with motor skills (hindlimb strength) on an inclined plane which involved the cerebellar functionality. Lower scores denoted poor cerebellar functionality by the Stx2-treated animals in comparison to their controls. The most significant lower score was obtained in the higher dose group (1 ηg) after five days of toxin administration (**Figure 2**). The control group (vehicle) showed the best performance in the task (highest score), when compared to the animals treated with 0.5 or 1 ηg of Stx2.

### Stx2 Caused Profound Ultrastructural Alterations in Purkinje Cells and Granular Layers

**FIGURE 3:**
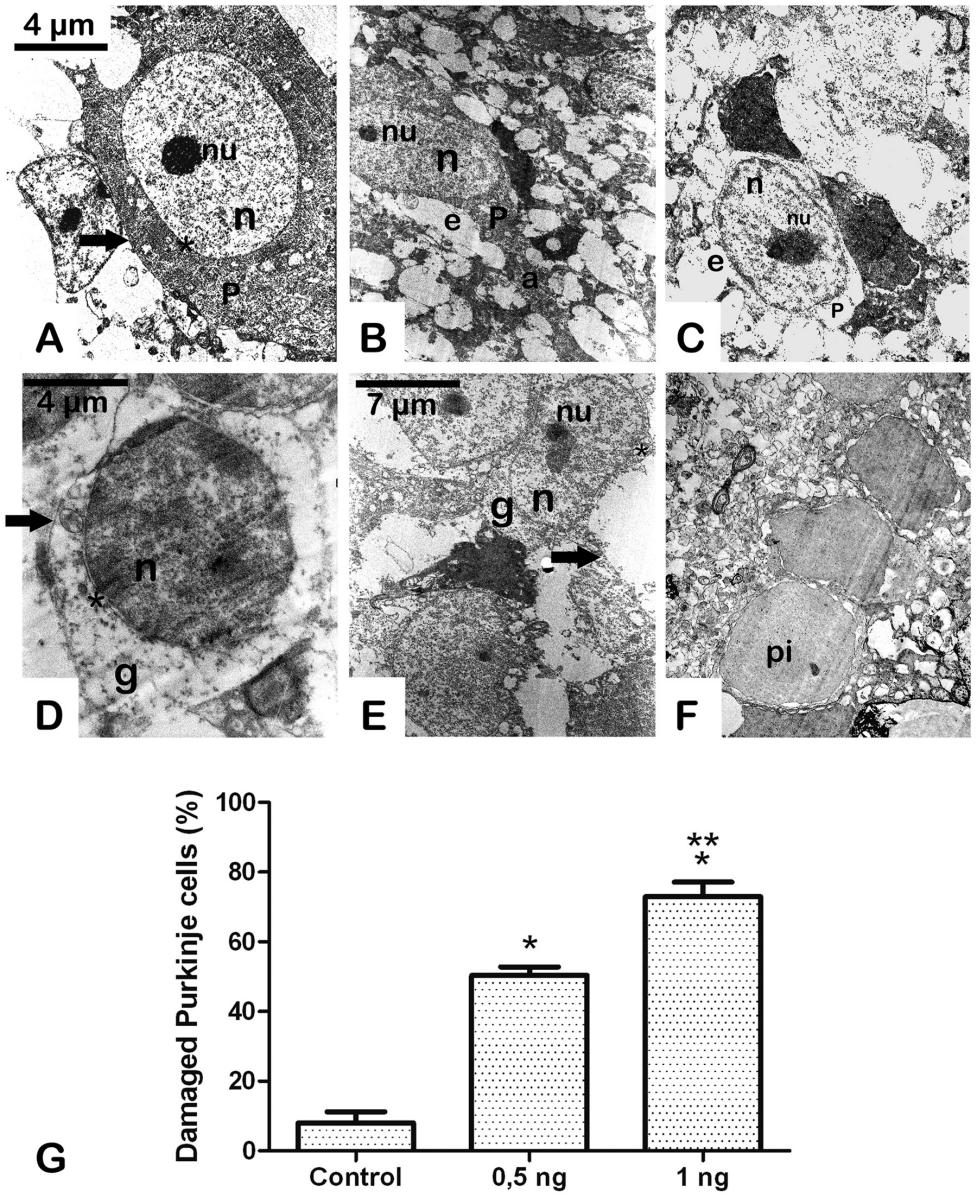
**A Sub-lethal dose of Stx2 causes cell damage in the cerebellum: dose response damage in Purkinje cells (A–C).** dose response damage in cells from the granular cell layer **(D–F)**. Vehicle **(A,D)**. Treatment with 0.5 ηg of Stx2 **(B,E)**.Treatment with 1 ηg of Stx2 **(C,F)**. Quantification of the percentage of damaged neurons **(G)**. Results are expressed as a percentage of the total number of neurons in an area of 3721 μm^2^. Significant differences between toxin-treated and control groups (^∗^*p* < 0.05). Significant differences between 1 ηg toxin-treated and 0.5 ηg toxin-treated groups (^∗∗^*p* < 0.05). nu, nucleolus; n, nucleus; P, Purkinje cell; e, edema; a, axon; pi, pycnotic cell; g, granular cell; asterisk, cell membrane; arrow, cell membrane. The scale bar in **(A)** applies to micrographs **(B)** and **(C)**. The scale bar in **(E)** applies to micrograph **(F)**.

Conserved Purkinje cells of the cerebellum from the vehicle-treated group showed visible pale nuclei and well-dispersed chromatin, intact cytoplasmic and nuclear membranes, and intact cytoplasm (Bishop et al., 1980) (**Figure 3A**). In contrast, neurons from the Stx2-treated group showed a progressive neurodegenerative condition in a dose-dependent manner (**Figures 3B,C,G**) compared to the vehicle-injected group (**Figure 3A,G**). Five days after administration of 0.5 ηg of Stx2 the nuclei of Purkinje cells started to become shrunk, eccentric and with edema (**Figure 3B**). In addition the bulk of chromatin was more condensed. Cytoplasms became vacuolated and electron-dense, and the axons were hypertrophic (**Figure 3B**). In addition to this, granular cells from the granular layer displayed similar ultrastructural alterations: nuclear edema, discontinuous nuclear membrane, vacuolated and shrunken cytoplasm (**Figure 3E**) a condition found in models of severe encephalopathies (Yu et al., 1971). This was not observed in granular cell layer from vehicle-treated-animals (**Figure 3D**). When Stx2 was administered more concentrated (1 ηg) a more profound ultrastructural alteration that resembled a more neurodegenerative condition was observed. Purkinje cells were found with abundant edema and retracted cytoplasm (**Figure 3C**). Also, at this amount of toxin, cells from the granular layer become shrunk, vacuolated and with necrotic appearance (**Figure 3F**).

## Discussion

Motor behavioral alterations assessed by the inclined plane test may be related to damage of cerebellar cells. In this report, the deleterious effect of sublethal intravenous Stx2 in the cerebellum was demonstrated using behavioral, ultrastructural, and physiological methodologies. Therefore, the current results mimic cerebellar compromise usually reported in patients with neurological manifestations derived from HUS (Hager et al., 1999; Steinborn et al., 2004; Donnerstag et al., 2012).

The inclined plane task is a behavioral test that reveals a cerebellar sensory-motor compromise. It tests the animal’s ability to maintain its position and thus can be used as an index of hindlimb strength (Fehlings and Tator, 1995), and for determining cerebellar functionality in experimental animal models of cerebellar lesions like cerebellar ataxia and cerebellar hemorrhage (Fehlings and Tator, 1995; Wells et al., 2003; Chang et al., 2008; Lekic et al., 2011). In addition, the integrity of pyramidal and extrapyramidal systems may also be considered in the performance of the task since peripheral Stx2 alters the functionality of these structures as we previously described (Pinto et al., 2013; Tironi-Farinati et al., 2013). The treatment of a sub-lethal administration of Stx2 caused low performance in the inclined plane task, in a dose response manner, during the early period of assessment in comparison to controls.

MRI studies in children with diarrhea associated to HUS showed nonspecific abnormalities in the cerebellum and these studies failed to show any correlation between the onset of neurological symptoms and MRI visible lesions (Donnerstag et al., 2012). Furthermore, the extent of these lesions did not correlate with the severity of neurological symptoms (Weissenborn et al., 2012). In contrast to this, peripheral or central administration of Stx2 succeeded to show by TEM deep alterations of the encephalon at the ultrastructural level (Fujii et al., 1996, 1998, Goldstein et al., 2007). Moreover, in the present work we certainly found a correlation between the altered ultrastructure and the behavioral and physiological abnormalities observed in toxin-treated animals. Therefore, the TEM technique constitutes a reliable tool to observe evident ultrastructural alterations in the cerebellum not detected by clinical routine imaging techniques.

In a previous work, we found a neurodegenerative ultrastructural phenotype in the brain striatum of mice (Tironi-Farinati et al., 2013). In the present work we observed similar findings in the cerebellum of mice. Sub-lethal Stx2 administration showed deep signs of neuronal damage. Purkinje and granular cells showed a degenerative condition in a toxin-dose response manner leading to cell death and this may occur through Gb3 receptor. It has been reported that Stx2 binds to Gb3 receptor in neurons, including Purkinje and granular cells in the cerebellum (Obata et al., 2008). Cerebellar cells in neurodegenerative conditions associated with disease were frequently found in animal models (Sato et al., 2012) or by drug injury (Sobaniec-Lotowska, 2001). Overall, the altered changes observed in these neurons reflect a pathological condition in Stx2-treated mice. The precise cell mechanisms involved in the neuropathology are still unknown and are under current investigation.

Other subtypes of Stx2 have been reported to produce neurological compromise. It has been reported that Stx2c and Stx2d caused damaged in the central nervous system (neuronal and endothelial ultrastructural alterations) and neurological symptoms (encephalopathy syndrome) in mice experimental models (Fujii et al., 1994; Amran et al., 2013), similar to Stx2a as it was observed in the present work and in a previous study by our group (Tironi-Farinati et al., 2013). In addition Stx2e induced the breakdown of the BBB and neurological disturbance in a porcine model (Meisen et al., 2013), a similar finding to our results.

In light of conclusive data presented, sub-lethal Stx2 altered the BBB permeability in the cerebellum. This event allowed the toxin to penetrate the cerebellar parenchyma that led to the observed cell damage. Previous data presented by us and by other colleagues demonstrated the capacity of the toxin to cross the BBB in other brain areas (Mizuguchi et al., 1996; Fujii et al., 1998; Goldstein et al., 2007). However, this is the first report acknowledging BBB permeability by the toxin in the cerebellum. This experimental model allows us to explain the cerebellar syndrome described in patients with HUS and may provide the basis for future research in prevention of neurologic damage.

## Author Contributions

All authors listed, have made substantial, direct and intellectual contribution to the work, and approved it for publication.

## Conflict of Interest Statement

The authors declare that the research was conducted in the absence of any commercial or financial relationships that could be construed as a potential conflict of interest.

## Abbreviations

BBB: blood–brain barrier
HUS: Hemolytic–Uremic Syndrome
Stx 2: Shiga toxin 2
TEM: transmission electron microscopy
